# Transformational improvement in quality care and health systems: the next decade

**DOI:** 10.1186/s12916-020-01739-y

**Published:** 2020-10-29

**Authors:** Jeffrey Braithwaite, Charles Vincent, Ezequiel Garcia-Elorrio, Yuichi Imanaka, Wendy Nicklin, Sodzi Sodzi-Tettey, David W. Bates

**Affiliations:** 1 Centre for Healthcare Resilience and Implementation Science, Australian Institute of Health Innovation, Macquarie University, Level 6, 75 Talavera road, North Ryde, NSW 2113 Australia; 2 Department of Experimental Psychology, University of Oxford, Oxford, OX2 6GG UK; 3 Institute for Clinical Effectiveness and Health Policy (IECS), Viamonte 2146 – 3 Piso, C1056ABH Ciudad de, Buenos Aires, Argentina; 4 Graduate School of Medicine and Faculty of Medicine, Yoshida-Konoe-cho, Sakyo-ku, Kyoto University, Kyoto, 606-8501 Japan; 5 International Society for Quality in Health Care (ISQua), Huguenot House, Dublin, D02 NY63 Ireland; 6 Institute for Healthcare Improvement, Boston, MA 02109 USA; 7 Harvard Medical School, Boston, MA 02115 USA

**Keywords:** Health systems, Patient safety, Quality of care, Universal health coverage, Low-, middle- and high-income countries

## Abstract

**Background:**

Healthcare is amongst the most complex of human systems. Coordinating activities and integrating newer with older ways of treating patients while delivering high-quality, safe care, is challenging. Three landmark reports in 2018 led by (1) the Lancet Global Health Commission, (2) a coalition of the World Health Organization, the Organisation for Economic Co-operation and Development and the World Bank, and (3) the National Academies of Sciences, Engineering and Medicine of the United States propose that health systems need to tackle care quality, create less harm and provide universal health coverage in all nations, but especially low- and middle-income countries. The objective of this study is to review these reports with the aim of advancing the discussion beyond a conceptual diagnosis of quality gaps into identification of practical opportunities for transforming health systems by 2030.

**Main body:**

We analysed the reports via text-mining techniques and content analyses to derive their key themes and concepts. Initiatives to make progress include better measurement, using the capacities of information and communications technologies, taking a systems view of change, supporting systems to be constantly improving, creating learning health systems and undergirding progress with effective research and evaluation. Our analysis suggests that the world needs to move from 2018, the year of reports, to the 2020s, the decade of action. We propose three initiatives to support this move: first, developing a blueprint for change, modifiable to each country’s circumstances, to give effect to the reports’ recommendations; second, to make tangible steps to reduce inequities within and across health systems, including redistributing resources to areas of greatest need; and third, learning from what goes right to complement current efforts focused on reducing things going wrong. We provide examples of targeted funding which would have major benefits, reduce inequalities, promote universality and be better at learning from successes as well as failures.

**Conclusion:**

The reports contain many recommendations, but lack an integrated, implementable, 10-year action plan for the next decade to give effect to their aims to improve care to the most vulnerable, save lives by providing high-quality healthcare and shift to measuring and ensuring better systems- and patient-level outcomes. This article signals what needs to be done to achieve these aims.

*We need health systems that enable the provision of higher-quality and safer healthcare, reflect population needs, and facilitate better assessment and management of population health. Three major reports in 2018 argue for such systems transformation* [1–3]*. Can it be achieved?*

## Background

The organisation and provision of healthcare is changing. Looking back over the centuries, physicians and other healthcare professionals had rudimentary practices, patients had modest expectations and there was no real health system as clinicians were independent providers. However, by the late twentieth century, healthcare had evolved to become the most complex of human endeavours [4]. The range of patient types and conditions, procedures, tests, technologies available, drugs, specialist workers and settings in which care was delivered, even in low- and middle-income countries (LMICs) with modest resources, grew exponentially [5, 6]. This demanded extensive supporting structures and coordination.

Fast forward to the third decade of the twenty-first century. We now have sophisticated information and telecommunications technologies, genomics, precision medicine, artificial intelligence, advanced pharma and unrivalled devices to support care, all of which are adding more capacity to deliver better treatment and interventions [7]. While LMICs have some access to these, they must do things less expensively—for example, by using mobile technology and microgrids rather than large computer systems. These new additions to the caring environment confer benefits to patients yet are immeasurably increasing systems complexity [8], and accelerating the need to be even better at coordination so all in the healthcare orchestra sing from the same song sheet.

### Major reports in 2018

Three recent milestone contributions address the issue of quality of care primarily as it affects the developing world yet with key messages for all health systems, developing or mature [1–3]. They offer recommendations for how to create better delivery systems and improve care quality. They each aspire, amongst all this complexity, to put care on an improvement gradient—to make it safer, of higher quality, and more responsive to demands across time. They also suggest that systems are becoming tougher to manage and coordinate, and enhancement of care and care settings, from policy to management to practice, is very hard to achieve. In this article, we analyse these reports individually and as a whole, and, as Academicians of the International Academy of Quality and Safety (IAQS) established by the International Society for Quality in Health Care (ISQua), we aim to describe the commonalities and differences and establish the platform from which we can improve care internationally. We begin with a synthesis of the main thrust of the arguments in the three reports.

The Lancet report (Box 1) makes a set of recommendations for how national governments and funding partners can take the lead in creating better, more caring, less harmful systems. It argues that a core enabler for achieving these goals will be a research agenda to underpin the key advances which need to be achieved (see Box 1 for a summary).

The WHO/OECD/World Bank report (Box 2) envisages widespread enabling action by governments, health systems and citizens, patients and health workers. It suggests that these bodies and groups should be encouraged to collaborate and conspire to deliver improved care across-the-board (see Box 2 for a summary).

The National Academies’ report (Box 3) represents a clarion call for more research, better measurement and concerted international efforts. It suggests creating a learning health system which contrasts with today’s forgetting system, and encourages people to think in systems terms rather than in silos or episodes (see Box 3 for a summary).

## Method

To understand more deeply the tenor and extent of the reports, we conducted an analysis of them using text-mining software provided by Leximancer 4.51, an automated content analysis computer program [9, 10]. Leximancer facilitates the interrogation of texts—words, and word segments—and creates a thesaurus of significant terms, known as textual concepts. The software allows the examination of the connectivity and relationship between concepts in large text documents. Groups of the most highly connected concepts are referred to as themes. Leximancer produces a ranked list of themes and a visual map of their connections, providing details of both the strength of a concept as it recurs within the text and the extent of connections to other concepts. Leximancer’s automated interrogation of documents can be applied to PDF and word processed documents. Essentially, the Leximancer program breaks down textual material into its key categories (themes, concepts) in order to visualise and quantify the text [10, 11].

## Results

Figures 1, 3, 5 and 7 provide the Leximancer maps of themes and concepts, accompanied by the frequency of their highest recurring themes (Figs. 2, 4, 6 and 8).

**Fig. 1:**
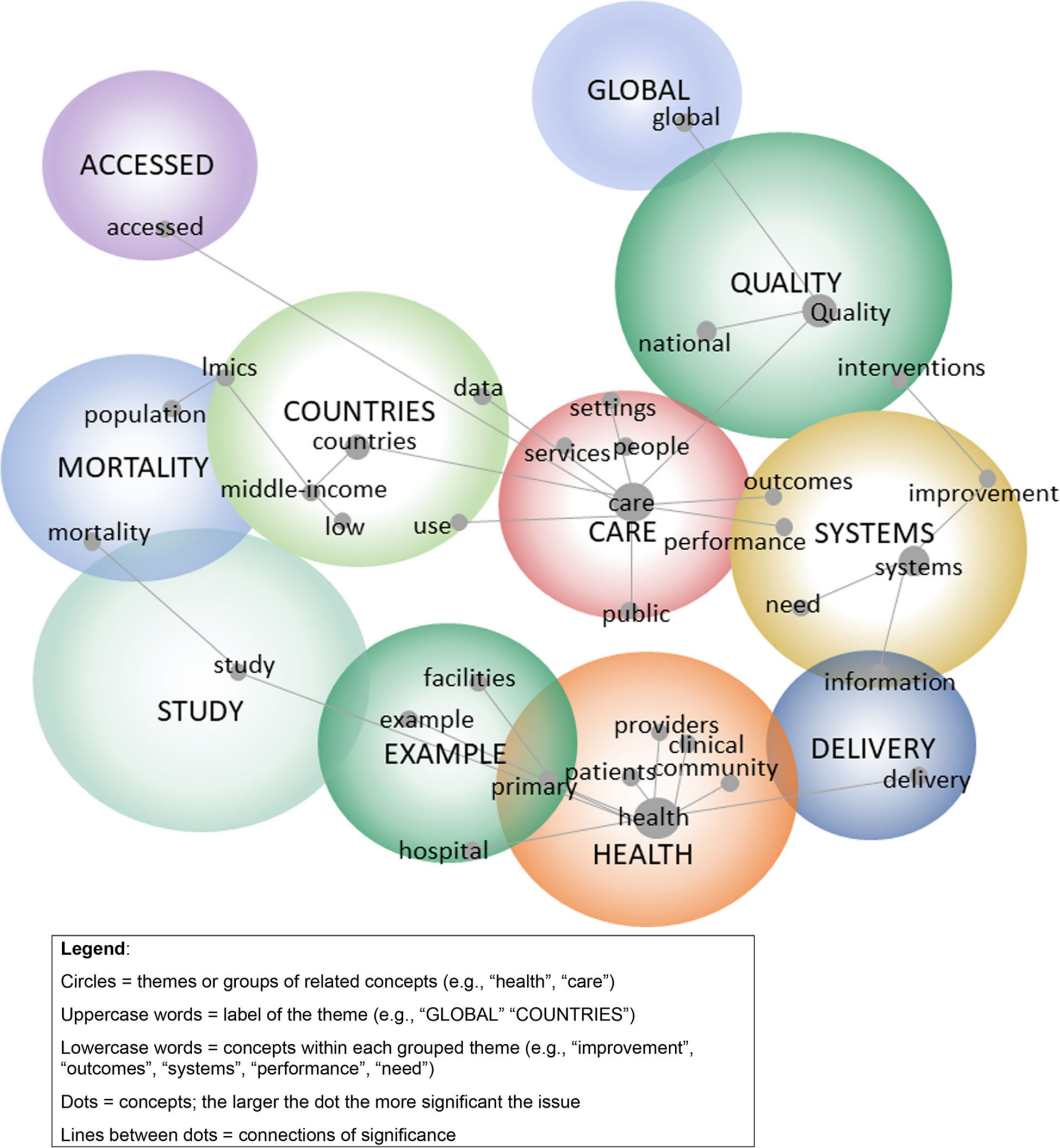
Synthesis of three reports—themes and concepts

**Fig. 2:**
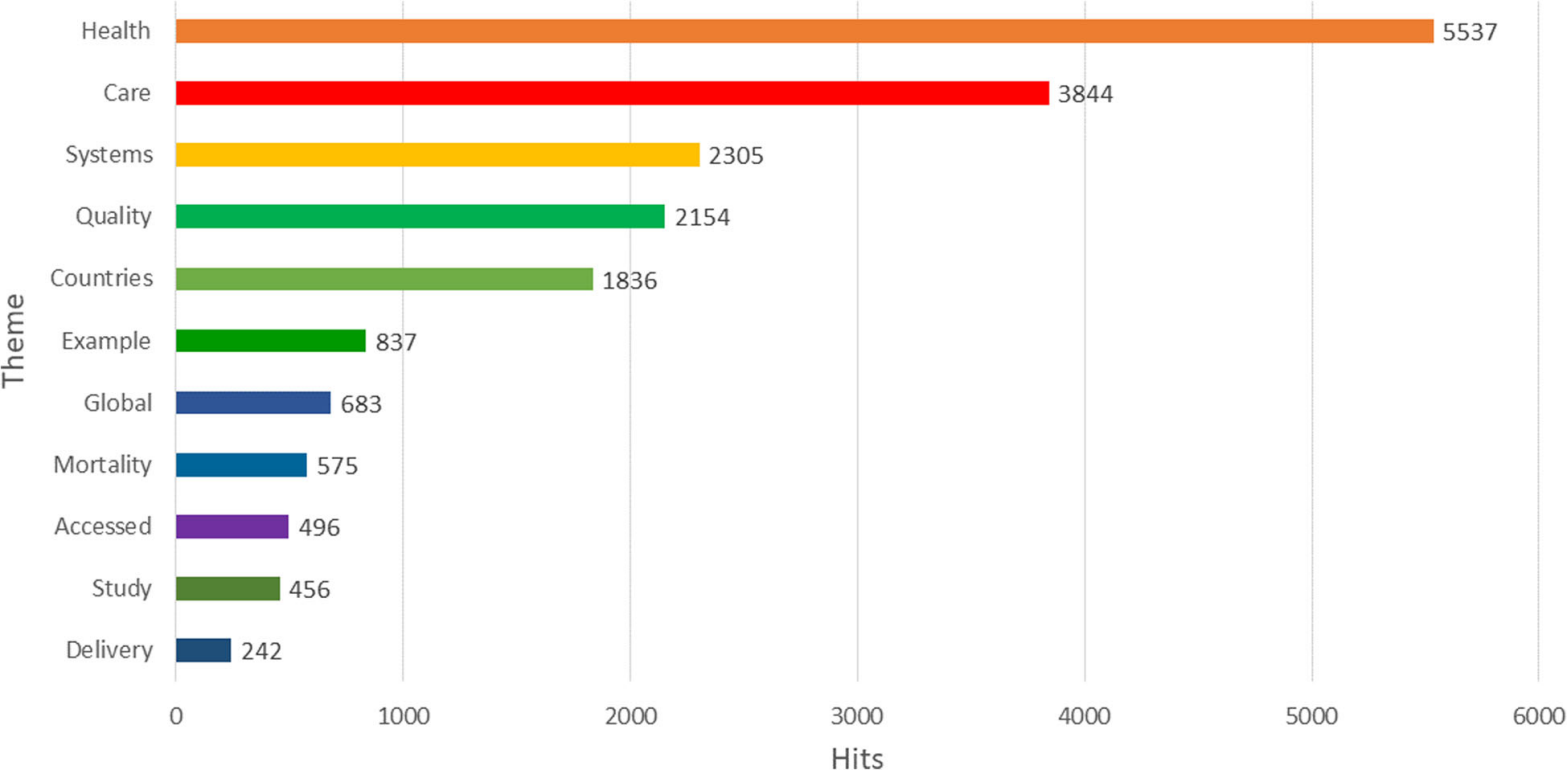
Synthesis of three reports—theme frequency summary

**Fig. 3:**
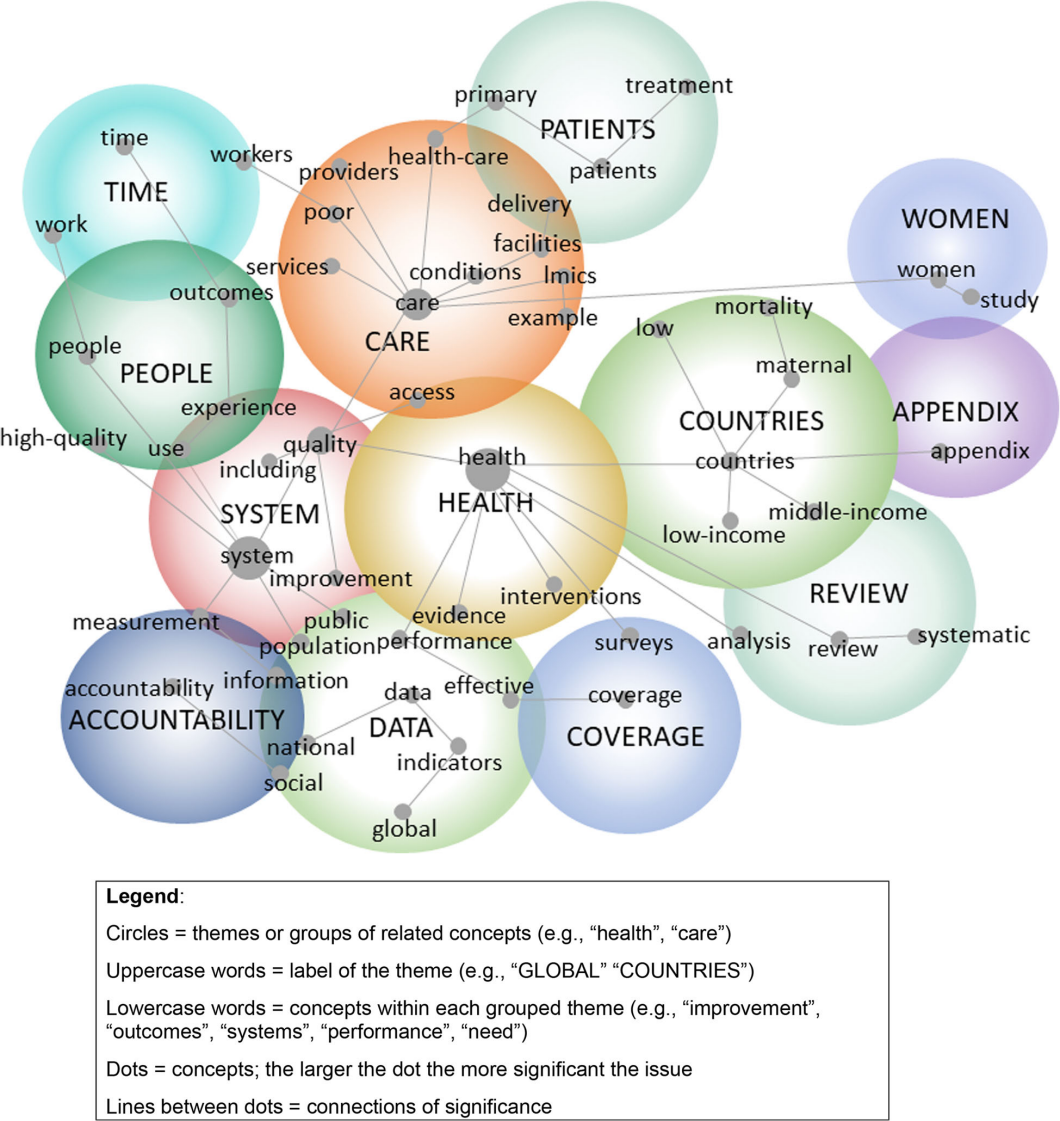
Lancet report—themes and concepts

**Fig. 4:**
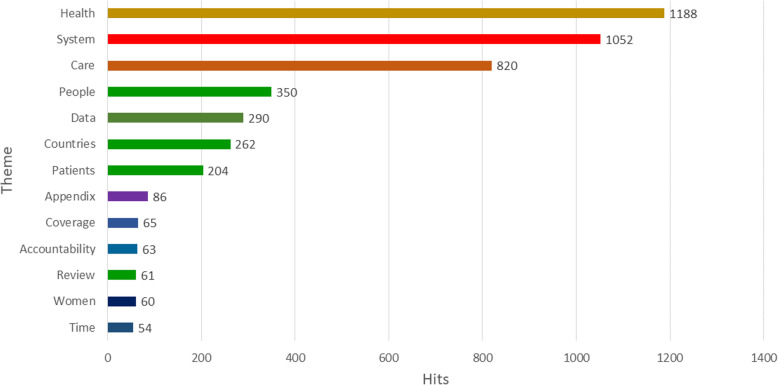
Lancet report—theme frequency summary

**Fig. 5:**
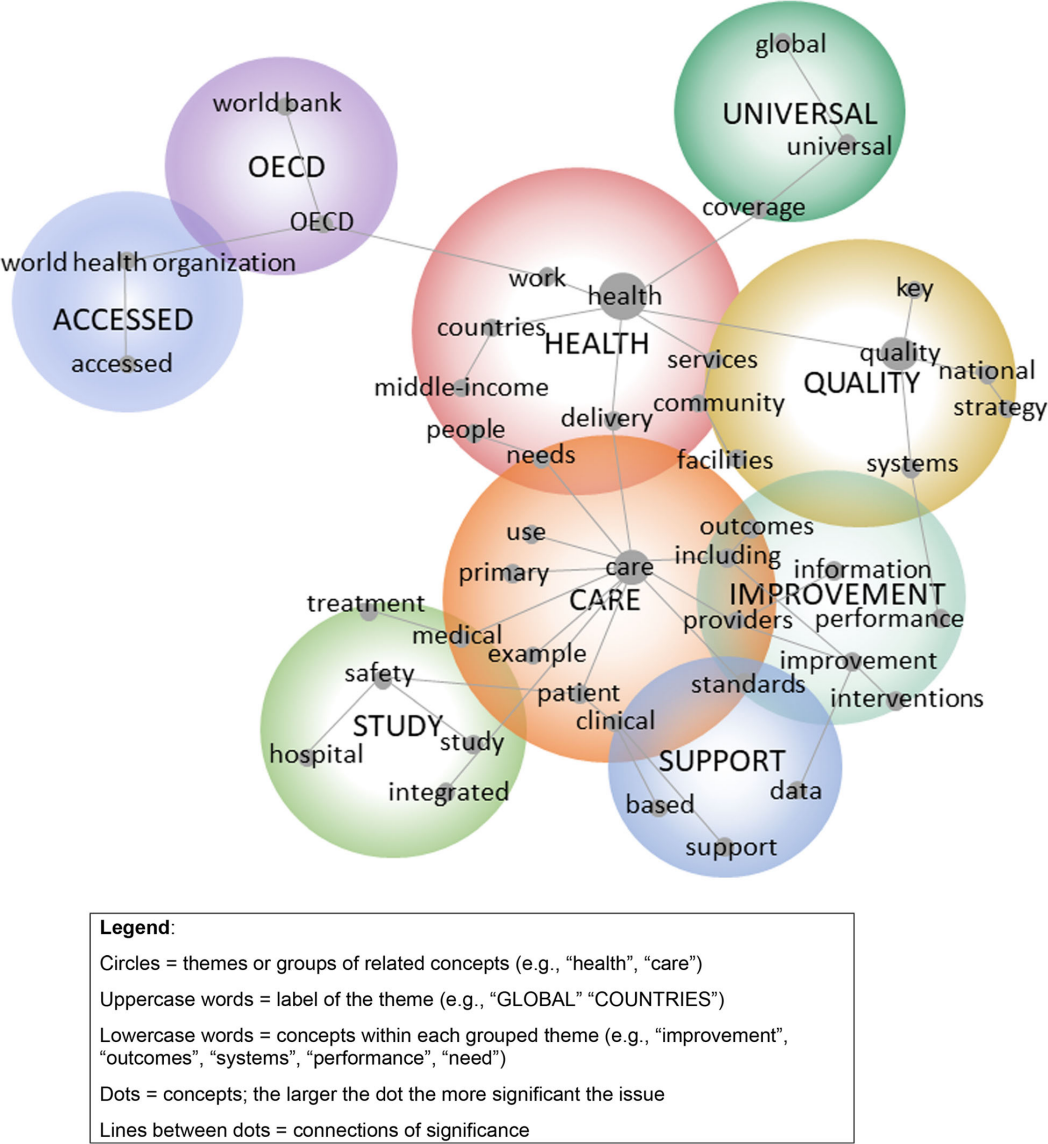
WHO/OECD/World Bank report—themes and concepts

**Fig. 6:**
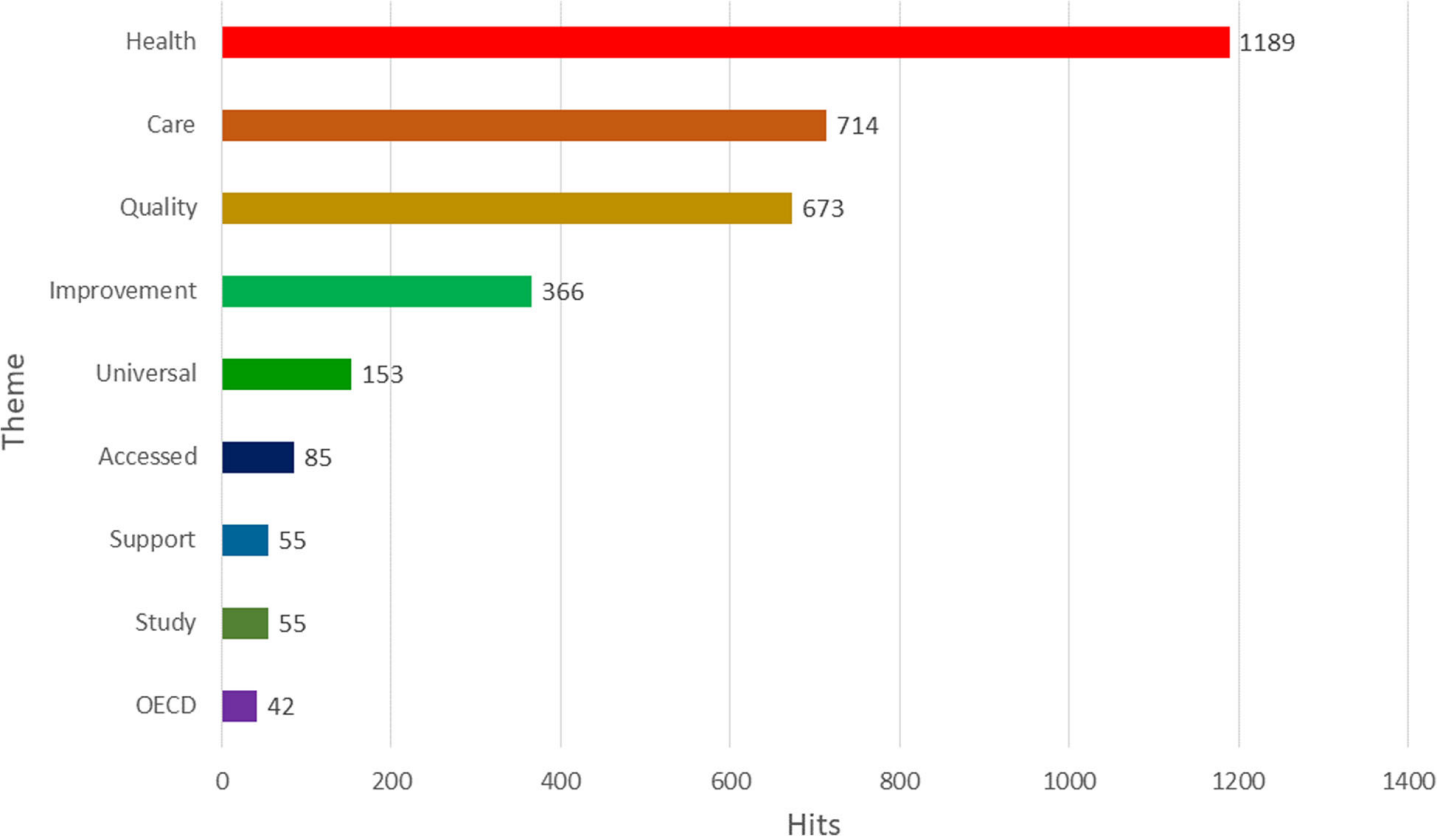
WHO/OECD/World Bank report—theme frequency summary

**Fig. 7:**
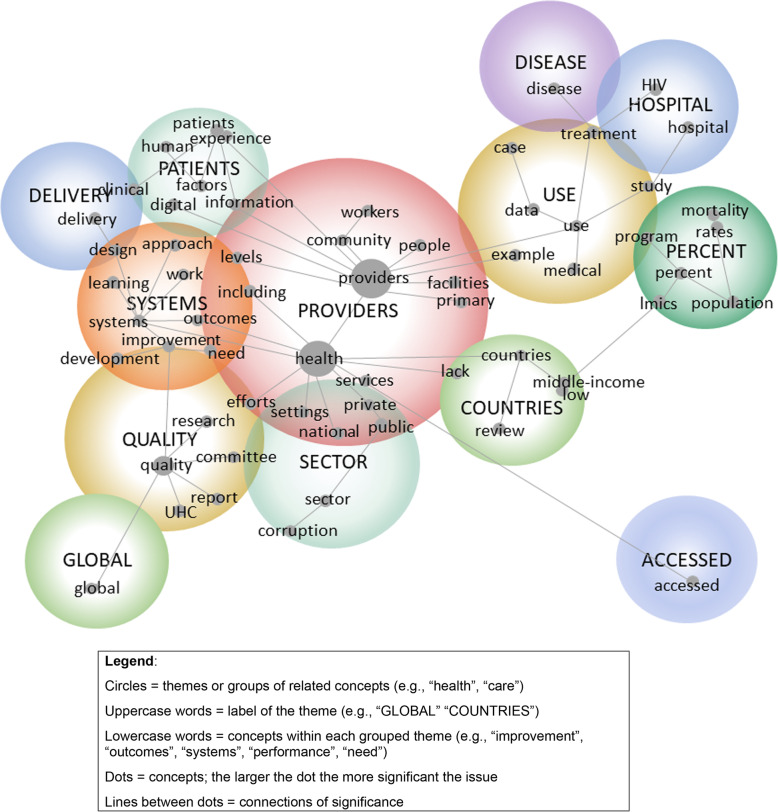
National Academies of Sciences, Engineering and Medicine report: Crossing the Global Quality Chasm: Improving Health Care Worldwide—themes and concepts

**Fig. 8:**
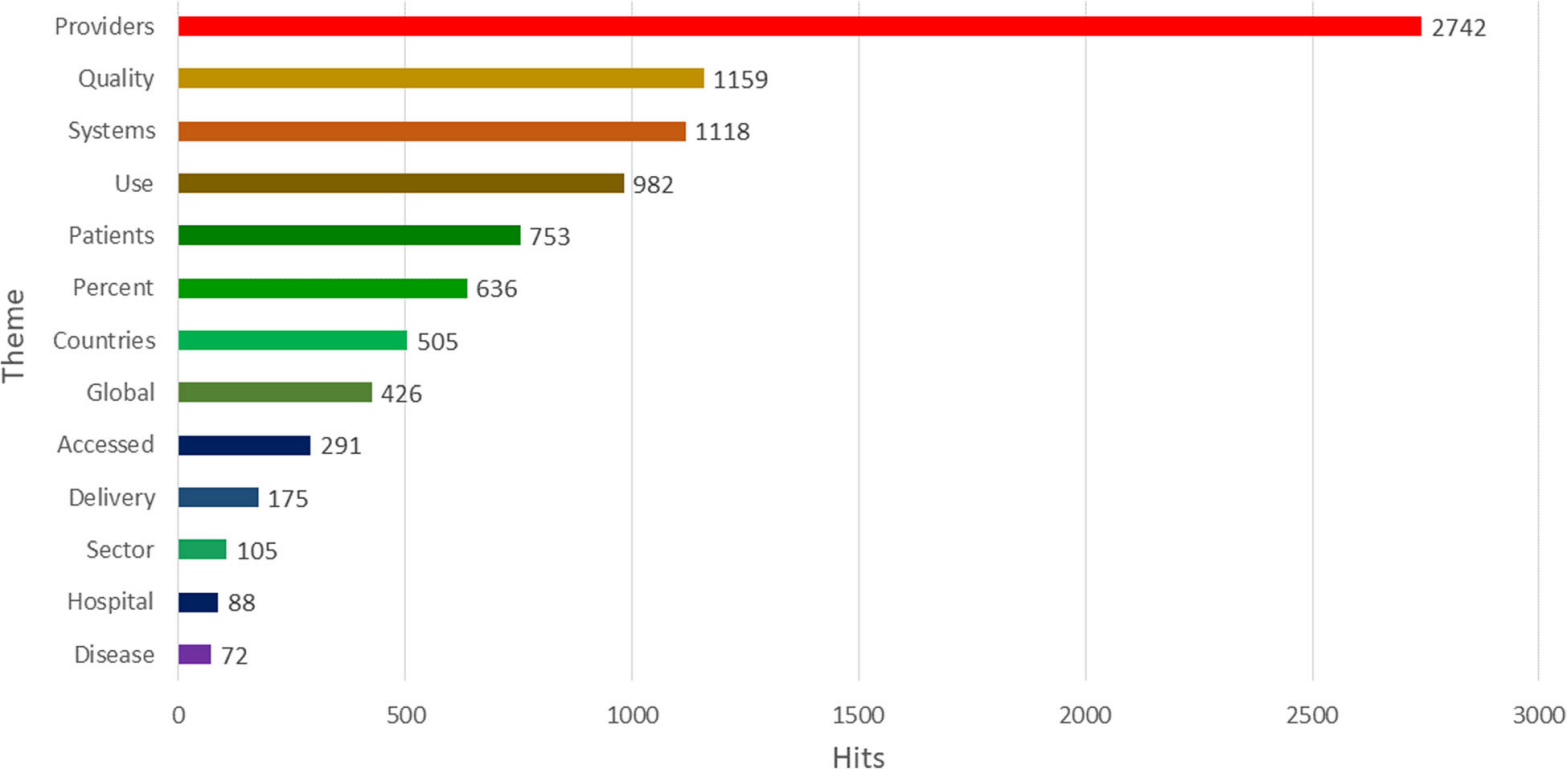
National Academies of Sciences, Engineering and Medicine report—theme frequency summary

In the first visual diagram (Fig. 1), eleven themes (circles) represent the content of the three reports in broad outline (see Fig. 2 for theme frequency summary of Fig. 1).

Across the three reports, “health”, “care”, “systems”, “Quality”, “countries” and “examples” are the most frequently occurring themes. These key themes suggest the reports are largely focused on the quality of care provided by health systems within and across various countries, with multiple examples provided. The graphical representation of Fig. 1 depicted in Fig. 2 shows the strength of the recurrences (“hits”) of each theme.

In Fig. 3, thirteen themes (circles) represent the content of the Lancet report in more detail (see Fig. 4 for theme frequency summary of Fig. 3).

Over the pages of the Lancet report, “health”, “system”, “care”, “people”, “data” and “countries” are the most frequently occurring themes. These key themes show how the report is mostly focused on people and data in healthcare systems across all countries of the world.

In Fig. 5, nine themes (circles) represent the content of the WHO/OECD/World Bank report in more detail (see Fig. 6 for theme frequency summary of Fig. 5).

Across the WHO/OECD/World Bank report, “health”, “care”, “quality”, “improvement”, “universal” and “accessed” are the most frequently occurring themes. These key themes suggest the report is mostly focused on the improvement of quality of care and access to care, particularly universal health coverage.

In Fig. 7, thirteen themes represent the content of the National Academies’ report in more detail (see Fig. 8 for theme frequency summary of Fig. 7).

For this report, “providers”, “quality”, “systems”, “use” and “patients” are the most frequently occurring themes. These key themes suggest the report is focused more on providers of care than are the other reports and recommends they offer quality care and effective services to users of health systems.

## Discussion

### The reports in context

Both the Leximancer automated content analyses and our boxed summaries demonstrate that there are more commonalities than differences running through the reports. One intriguing question is why three extraordinarily influential reports in the same domain saw the light in the same year? There are plenty of historical examples of an idea whose time has come. It seems that the thrust of the reports, and their confluence, may signal we have arrived at a crossroads, and the time is ripe to expedite transformational efforts.

Particularly important areas of convergence in these documents are the idea of universal coverage and raising the bar on quality of care and of health systems performance so all citizens in all countries benefit, especially those in the poorest regions. Instead of seeing healthcare in silos (acute settings, community care, general practice, aged care) or in terms of clinical conditions, disease types or specialities (malaria, diabetes, paediatrics), the reports seek coordination and integration of care across the continuum, centred on patients’ rather than providers’ needs. The reports urge countries to provide incentives in the right place to improve care for best effect. They do not shy away from how complex an undertaking healthcare delivery is and each argues that taking a systems view to problems is critical.

Strategies the reports discern as most useful for improvement include better leadership and governance, judicious and cost-effective use of proven ICT, more training and education for workforces, and applying what is already known, such as via the use of clinical practice guidelines, safe surgical checklists and vaccinations across entire populations for herd immunity. The reports’ authors advocate for a patient-based rather than a provider-focused perspective and want those across caring systems to be better at implementation science, translation and disseminating the success exhibited in one setting to others, at scale. Each argues for the importance of measuring progress longitudinally, conducting well-designed evaluation to learn from what is working and what is not, and underpinning improvement efforts with a suitably resourced, purpose-designed research program.

As to differences between the reports and their recommendations, these are surprisingly few, and a matter of degree. *The Lancet* report focuses on health systems and is more data-rich and research-intensive (there are 327 references cited) and it hones in on saving lives, equity of access and care, measurement of outcomes and transformation of current care systems. The WHO/OECD/World Bank volume links quality improvement to universal health coverage and makes recommendations to four stakeholder groups (governments, health systems, citizens and patients, and healthcare workers), seeking to enrol these groups in the work ahead. Authors of *Crossing the Global Quality Chasm* are focused on the six dimensions of quality (safety; effectiveness; person-centredness; accessibility, timeliness, affordability; efficiency; and equity), and take a systems perspective on change, establishing agreed principles for systems redesign and seeking to harness digital progress across countries. They also mount strong arguments for change proponents to focus on informal care and carers, and to reduce corruption.

They all note that poor-quality care remains an entrenched, wicked problem. All-in-all, collectively, the authors of the three volumes challenge those presiding over every health system to lift their game and do better for the benefit over the medium to long term of billions of people.

### Calling for international action

How can we move from 2018, the publication year of the reports, to 2020 and beyond, as the decade of action? Going beyond the extensive learnings within these documents, we argue for three calls to action.

### Call to action 1: establish a 10-year blueprint for change

The combined recommendations of the reports will doubtless shape the work of the international community over at least the next 10 years. Yet a very real concern we have is that too often with almost all authoritative reports on health systems reform, whether national, regional or global, much effort is expended on conducting the studies and formulating the recommendations and then the study task force membership dissolves and individuals go back to their normal activities. Less attention is later given to how recommendations might be implemented, translated into learning and adoption strategies, and for political and policy impetus for change to match the importance of the recommendations. We hesitate to call for yet another report to document how to put the recommendations into action; however, we need a blueprint for change and a plan to measure progress. This would articulate, as everything cannot be done simultaneously, the optimal stages to be followed in addressing all the change suggested by the reports, who should do what and with what levels of accountability, and how the interactions between stakeholders will play out. We also need a much clearer idea of how the international community and individual health systems might design rigorous evaluation studies to assess progress and feed that progress back to stakeholders in the system responsible for the improvements. Overall, we must find better ways to document how to disseminate best-practice models, and to achieve improvement at scale, and incorporate these in the blueprint for 2030.

### Call to action 2: buy more equitable, high-quality care for the greatest number

Second, collectively the reports plead for every country to move along an improvement gradient; implementation is proposed as a country-level responsibility but with a global commitment, supported by international bodies such as the UN’s agencies, the WHO and OECD. So, let us take the word “global”, which each of the three contributions uses emblematically and often, seriously. Say we wanted to do the very best we can for the 7.7 billion people in the world, taking what these reports have said to heart, and as a broad framework for change. What is that “very best we can”? It will need to be something that would galvanise the international community and seek to create sufficient leverage for major step-changes in the healthiness of the world.

It seems to us that if we wanted to buy the greatest amount of high-quality care we could, do the most good for the most people and maximise reductions in harm, we would find ways to genuinely divert tangible allocations of money to low- and middle-income health systems. That single action (as hard as it might be to accomplish, as it would mean generating or transferring substantial amounts of funding from the budgets of wealthy countries to less wealthy populations) would reduce inequalities and buy more health gain for more people at the least cost than continuously increasing the spending of GDP on rich health systems already consuming between 10 and 17% of national income such as the USA, Japan, France and Canada. If we did this, at a stroke we would provide more life-saving, life-enhancing initiatives and could share the advantages of higher-quality care, institute training in continuous improvement, create best-practice patient safety and even mobilise the potential benefits of artificial intelligence in medicine, genomics and modern ICT, to many more people. This could be promoted as an idea whose time has come. Senator Elizabeth Warren of Massachusetts, a prominent United States (US) Democrat who was pitching in 2019 for the 2020 presidential nomination of her party, estimated that just 2% annual tax on US families with fortunes of over US$50 million would generate US$2.75 trillion over a decade [12]. As this would capture sufficient funding for US student debt forgiveness, and as well two thirds of all US students would receive free college tuition for this amount, imagine the good that could be done if the richest OECD countries did the same and diverted even 1% annual tax on the wealthy to low-income health systems. We would perhaps generate US$10 trillion, which could be put to use, saving lives, providing more care, improving the health of the world and reducing disparities. This would be politically difficult of course, but not impossible.

But even if we did not go this far, by way of illustration, we modelled how much we could generate and potentially divert by allocating just 1%, not of annual tax, but merely of health spending in five wealthy countries, creating a pool of almost US$50 billion (Table 1) [13, 14]. We then looked at nine lower-income countries in Africa, Asia and the middle-East and examined the costs of selected programs currently being advanced to make life better, improve quality of care or just to make life bearable (Table 2; synthesised from GlobalGiving) [15]. The costs are so modest compared with the pool of US$50 billion that it is virtually impossible to argue against the proposition that we must do something urgently and sustainably to address this level of inequity in some form or another. For so little cost, so much could be gained.

**Table 1:** Wealth generated from 1% of per capita health spending in five selected OECD countries [13, 14]

High-Income Country	Health spending (US dollars/capita), per annum	1% of health spending in high-income countries (US dollars/capita), per annum	Population	Total ($US rounded)
USA	$10,586.08	$105.86	327,167,434	$34,633,944,563
Japan	$4,766.07	$47.66	126,529,100	$6,030,376,906
UK	$4,069.57	$40.69	66,488,991	$2,705,437,044
Germany	$5,986.43	$59.86	82,927,922	$4,964,065,411
Australia	$5,005.32	$50.05	24,992,369	$1,250,868,068
Total				**$49,584,691,992**

**Table 2:** Life-sustaining or quality-of-care-enhancing programs in nine selected lower-income countries [15–17]

Low-income Country [14]	Types of initiatives	Cost ($US) of program
Afghanistan	*Training midwives in Afghanistan.*	**$50,000** (e.g. $600 contributes to the development of three training modules to train 100 midwives each)
	As a preventative approach to the high maternal and infant mortality rates in Afghanistan, this initiative funds midwifery training to improve equity and access to essential women’s healthcare in rural areas.	
Burundi	*Providing healthcare to 1000s in Bujumbura slum.*	**$20,000** (e.g. $300 funds a 3-day trauma healing workshop for gender-based violence survivors)
	Funds healthcare support, trauma healing workshops and AIDS testing to the Ntaseka Clinic, for more than 5000 people per annum. Patients include survivors of gender-based violence and abuse, people who are HIV positive, and the general population.	
Chad	*Life skills and peer education for Chadian Youth.*	**$33,913** (e.g. $200 supports one community outreach even in N’Djamena)
	Funds quality programs to the Chadian youth; the programs which are peer-based, disseminate information about the risks of drugs, alcohol, and transmission of HIV/AIDS.	
Ethiopia	*Simple surgery to restore sight to Ethiopians.*	**$6000** (e.g. $12 funds the cost to conduct two eye surgeries to reverse the effects of blinding trachoma)
	Funds simple eye surgeries in Ethiopia, including remote Ethiopia, for those suffering from trachoma. The hope is to eradicate the eye disease.	
Liberia	*Restoring healthcare to women and girls in Liberia.*	**$100,000** (e.g. $1500 pays all clinic operating costs in Liberia for a month)
	Funds community-based preventative care and an acute care clinic in Kakata to women and girls in Margibi County, Liberia. The clinic provides emergency care and has over 200,000 patients.	
Malawi	*End mother to child transmission of HIV in Malawi.*	**$5000** (e.g. $25 can pay for 1500 text messages to support and educate adolescents on HIV and safe sex)
	This is the largest country program, and the first that employs and recruit’s HIV positive men as Expert Clients. There are over 270 Expert Clients in 98 clinics who support and educate other men in the area about HIV.	
Nepal	*Rescue children suffering from severe malnutrition.*	**$50,000** (e.g. $10 gives medicine to one child while they are restored to health at a Nutritional Rehabilitation Home)
	“Fifty percent of Nepali children under five-years-old are malnourished … Malnutrition is the main cause of death for as many as 50,000 Nepali children each year.” This program funds Nutritional Rehabilitation homes to restore the health of child and educate parents of the risks.	
Tanzania	*Make motherhood safe for Tanzanian women.*	**$400,000** (e.g. $1000 funds a C-section and recovery)
	Funds the construction, equipment and training of staff for a new maternity hospital in Dar es Salaam, Tanzania. There will be 22 facilities and a 200-bed hospital to improve maternal health and assist with decreasing the high maternal mortality rate in Tanzania.	
Uganda	*Stop Ugandan women and children dying at childbirth.*	**$36,000** (e.g. $4200 will buy one portable ultrasound scanning (Clarius) machine from the USA.)
	Funds maternal health and welfare in Uganda and increases the quality of care for women in childbirth through effective equipment, training of midwives, social workers and radiographers and supporting outreach programs.	

[Source: GlobalGiving [15]]

Add initiatives such as these to the new emphasis on integrity in the US’s National Academies report, and the need to fight corruption as a new dimension of quality. While we advocate for greater resources, we also call for greater integrity in its application, buttressed by more robust accountability mechanisms.

### Call to action 3: learn from what goes right as well as what goes wrong

Our third call to action leads with the point that all three reports start with the problems in the system: harm, lack of universality and poor-quality care, for example. What they do not recognise sufficiently is that we have not traditionally taken the trouble to learn very well from things going right and to develop ways of supporting this. This perspective, coming from scholars of resilient healthcare and Safety-II, asks not why do things go wrong, but in systems as complex as healthcare (and, as we have seen, in LMIC systems, as resource-deprived), how come so much goes right? Why do some providers, teams and microsystems deal capably with everyday challenges, producing good care despite other providers, teams and microsystems in the same system producing worse outcomes—yet they all face the same constraints? What workarounds are developed by staff to overcome system deficiencies? Lack of resources, poor support systems and less than optimal ICT are all perennial problems every system—even wealthy ones—faces. Traditionally, change agents and quality and safety advocates have been more interested in stamping out harm than extending understanding of what systems do in their entirety, good and bad, and how the work force adopts, coordinates and shares knowledge to keep patients safe and provide quality care. So running alongside the ideas and recommendations from these three reports, we argue we need exemplar studies of success—building, for instance, on the publication of recent case study exemplars [18]. Table 3 presents eight of these examples, by way of indicating the potential for implementable change at the country level, from a platform of prior successes. The Table shows how every country has something to offer other health systems in improvement and reform.

**Table 3:** Eight examples of country-level reform or improvement [7]

Country	Key indicators	Initiative	Success features	Outcomes
Argentina	Population: 44,494,502	**Implementation of various quality and safety initiatives** including the Categorising Authorization Program, National Program of Quality Assurance of Healthcare, and the National Program of Epidemiology and Hospital Infection Control	• Financing by World Bank and national and provincial governments	• Unification of licencing rules
	GDP per capita, PPP: $20,567.30		• External quality and patient safety evaluations	• Reduction of treatment variability
	Life expectancy at birth (both sexes): 76.7 years		• Contribution from specialised scientific societies	• Healthcare coverage for pregnancy, childbirth, postpartum care and paediatric care
			• Pay-for-performance strategy	
	Expenditure on health as a proportion of GDP: 7.5%		• Training initiatives on quality and patient safety	• Healthcare coverage for adolescents and women
	Estimated inequity, Gini Index: 40.6%			
Brazil	Population: 209,469,333	**Launch of Proqualis**—a website featuring relevant and current publications about Quality Improvement (QI) initiatives, social media platforms, and QI tools and strategies	• Organisation of website and editorial policy allowing for easy retrieval of information	• Provision of relevant and reliable QI information to consumers
	GDP per capita, PPP: $16,068.02			
			• Standardised terminology via a glossary of terms	• Increased access to QI information for health professionals and managers
	Life expectancy at birth (both sexes): 75.7 years			
			• Publications specify the relevance to the Brazilian context	
				• Improved access to tools and strategies to support QI for health professionals
	Expenditure on health as a proportion of GDP: 11.8%		• All materials are free to access	
			• Information accessible on tablets and mobile phones	
				• Increased communication through social media platforms
	Estimated inequity, Gini Index: 53.3%			
India	Population: 1.35 billion	**Introduction of Universal Health Coverage** through a public-private partnership model	• Support from private healthcare providers and insurance companies	• More accessible, affordable, safe and appropriate health services
	GDP per capita, PPP: $7761.60			
				• More than 50% of India’s population covered by health insurance
	Life expectancy at birth (both sexes): 68.8 years			
				• Financial protection to families living below the poverty line
	Expenditure on health as a proportion of GDP: 3.7%			
	Estimated inequity, Gini Index: 35.7			
Jordan	Population: 9,956,011	**Formation of the Health Care Accreditation Council**—a national healthcare accreditation agency	• Start-up funding from USAID	• Development of international accepted standards
			• Employee training though consultation and education departments	
	GDP per capita, PPP: $9347.94			• Development of health professionals’ capacity to improve quality and patient safety
	Life expectancy at birth (both sexes): 74.5 years		• Application of a total quality management philosophy to encourage sustainable change	
				• Increased use of family planning methods by clients
	Expenditure on health as a proportion of GDP: 5.5%			• More effective management of certain conditions, e.g. diabetes
				• Improved leadership commitment, employee involvement and teamwork
	Estimated inequity, Gini Index: 33.7			
				• Increased consumer satisfaction
				• Influenced the Ministry of Health to increase its Quality Department budget and personnel
Rwanda	Population: 12,301,939	**Establishment of Community-based health insurance**	• Nationwide initiative	• 90% coverage of population
			• Strong and sustained political commitment	
	GDP per capita, PPP: $2253.52			• Improved access to health services
			• Financial investment from the government	
	Life expectancy at birth (both sexes): 67.5 years			• Improvements in healthcare utilisation
			• Legislative support	
			• Consensus from the population that healthcare access should be equitable and affordable	• Reduction of financial catastrophe and impoverishment due to out-of-pocket costs
	Expenditure on health as a proportion of GDP: 6.8%			
			• Introduction of a stratification system based on individual assets	• Improvement of health indicators, e.g. reduced maternal mortality and under 5 years’ deaths
	Estimated inequity, Gini Index: 43.7			
Spain	Population: 46,723,749	**Advancement of the Spanish National Transplant Organization (ONT)**	• Existing legal, organisational and technical frameworks	• Increase in the number of patients receiving transplants
	GDP per capita, PPP: $40,854.58		• Coordination of donor activities at the national, regional and hospital level	• Increased organ donation rates
	Life expectancy at birth (both sexes): 83.3 years			• Highest deceased donation rates for a large country
			• Employment of transplant coordinators to facilitate identification and referral of possible donors	
				• Donation rates above that of the European Union or USA
	Expenditure on health as a proportion of GDP: 9.0%			
			• Training of professionals in organ donation	
			• Development of a positive public attitude towards organ donation though mass media and an open communications policy	
	Estimated inequity, Gini Index: 36.2%			
			• Hospital reimbursements for donations and transplantation activities	
Taiwan	Population: 23,508,428	**Adoption of health information technology**, e.g. USB-based electronic personal health record system, MyHealth Bank website, replacement of paper-based ID cards with smart card, and cloud-based systems	• Single-payer system	• Cost-effectiveness, e.g. reduction in administrative costs
			• Development of security mechanisms to protect consumers’ privacy and information	
	GDP per capita, PPP: $47,800			• More efficient, streamlined processes
	Life expectancy at birth (both sexes): 80.1 years			• Improved quality of information
				• Improved medication safety
	Expenditure on health as a proportion of GDP: 6.2%			
				• Enhanced collaboration and information transfer between providers
	Estimated inequity, Gini Index: 33.8%			• Unified public health and clinical medicine information systems
				• Engagement of consumers in their own care
				• Reduction of fraud
				• Continuity of care
West Africa (Guinea, Liberia and Sierra Leone)	Total population: 24,883,449	**Application of quality improvement efforts** in Ebola-effected countries	• Emphasis on recovery processes	• Knowledge sharing between
	GDP per capita, PPP: $1846.67*		• Systematic post-disaster needs assessments	• Ebola-affected countries
	Life expectancy at birth (both sexes): 58.61*		• Focus on infection prevention and control, and health worker protection	• ‘Global pool of knowledge’
				• Demonstration of a model to combat Ebola with application to other infectious diseases
	Expenditure on health as a proportion of GDP: 10.54%*		• Community input	
			• Clearly articulated vision for universal health coverage	
			• Strong leadership and guidance	
	Estimated inequity, Gini Index: 33.6*			

*Data averaged across Guinea, Liberia and Sierra Leone

All data are from the World Health Organization and World Bank. Available data used as at August 2019

*GDP* gross domestic product, *PPP* Purchasing Power Parity

We propose the creation of a database of successful case studies and learnings from things going right, built from the combined experience of the major international bodies (e.g. ISQua, WHO, World Bank, OECD). Specific country experiences with national- or regional-level programs, projects or interventions, with details of what works, for whom and under what conditions, would be of substantial benefit to others grappling with similar problems. And this takes us back to call to the first call to action—we need a persuasive blueprint for change, including allocating responsibilities for mining and using such information.

### Strengths and weaknesses of the calls to action

Although they are pitched at a high level, these three calls to action represent initiatives that, if adopted, could help galvanise the international community. The strengths of such a set of change ideas are that they are built from the reports themselves, and other international suggestions for change over the years [19–23]. As to weaknesses, it is recognised that the approaches we are suggesting may not receive sufficient attention in the present pandemic era. The current pandemic itself raises serious questions about health system resilience and the quality of Covid-19 and routine non-Covid-19 care. The Covid-19 pandemic amplifies the need for health system strengthening to address gnawing quality of care gaps that have intensified in the wake of the global crisis. Regarding the second call, rather than wait for resources to be released from the benevolence of rich countries, the Covid-19 pandemic teaches that it is time for resource-constrained countries to reprioritize health resource allocation. Ghana, for example, has demonstrated this through activation of local manufacturing of personal protective equipment.

That being said, development of a blueprint for action with a clear picture of what healthcare in 2030 should look like is possible; the funding required for call to action 2, despite the good it could bring to the world, may not be given priority by wealthy health systems; and call to action 3, more thoroughly understanding health systems from a systems standpoint and learning from things going right is increasingly receiving attention [24–28], yet has not yet become a mainstream idea. While these recommendations are for the long term, determination and a willingness to begin, even during a pandemic, are essential. Who has the courage to take the lead?

## Conclusion

If we actioned these calls to arms over the decade of the 2020s, and move the reports’ proposals into the real world with a template for change which synthesises and incorporates their recommendations, we might find ourselves on a path to progress in a strategic rather than piecemeal or fragmented way. And the potential benefits to humankind, of putting our money where our mouth is, galvanising the world’s expertise into an actionable plan, buying better care for less fortunate populations and more thoroughly understanding the whole caring enterprise, building on past success? These would be enormous.

## Data Availability

The datasets used and/or analysed during the current study are available from the corresponding author on reasonable request.

## Abbreviations

C-section: Caesarean section
GDP: Gross domestic product
HIV: Human immunodeficiency viruses
IAQS: International Academy of Quality and Safety
ICT: Information and Communications Technology
ID: Identity document
ISQua: International Society for Quality in Health Care
LMICs: Low- and middle-income countries
OECD: The Organisation for Economic Co-operation and Development
ONT: National Transplant Organization
PPP: Purchasing power parity
QI: Quality improvement
UHC: Universal health coverage
UN: United Nations
US: United States
USAID: United States Agency for International Development
USB-based: Universal serial bus based
WHO: World Health Organization
